# Differential synchrotron X-ray imaging markers based on the renal microvasculature for tubulointerstitial lesions and glomerulopathy

**DOI:** 10.1038/s41598-017-03677-x

**Published:** 2017-06-14

**Authors:** Yu-Chuan Lin, Yeukuang Hwu, Guo-Shu Huang, Michael Hsiao, Tsung-Tse Lee, Shun-Min Yang, Ting-Kuo Lee, Nan-Yow Chen, Sung-Sen Yang, Ann Chen, Shuk-Man Ka

**Affiliations:** 1 Graduate Institute of Life Sciences, National Defense Medical Center, Taipei, Taiwan; 2 Institute of Physics, Academia Sinica, Taipei, Taiwan; 3 Department of Radiology, Tri-Service General Hospital, National Defense Medical Center, Taipei, Taiwan; 4 Genomics Research Center, Academia Sinica, Taipei, Taiwan; 5 National Center for High-Performance Computing, Hsinchu, Taiwan; 6 Division of Nephrology, Department of Internal Medicine, Tri-Service General Hospital, National Defense Medical Center, Taipei, Taiwan; 7 Department of Pathology, Tri-Service General Hospital, National Defense Medical Center, Taipei, Taiwan; 8 Graduate Institute of Aerospace and Undersea Medicine, Academy of Medicine, National Defense Medical Center, Taipei, Taiwan

## Abstract

High resolution synchrotron microtomography capable of revealing microvessels in three dimensional (3D) establishes distinct imaging markers of mouse kidney disease strongly associated to renal tubulointerstitial (TI) lesions and glomerulopathy. Two complementary mouse models of chronic kidney disease (CKD), unilateral ureteral obstruction (UUO) and focal segmental glomerulosclerosis (FSGS), were used and five candidates of unique 3D imaging markers were identified. Our characterization to differentially reflect the altered microvasculature of renal TI lesions and/or glomerulopathy demonstrated these image features can be used to differentiate the disease status and the possible cause therefore qualified as image markers. These 3D imaging markers were further correlated with the histopathology and renal microvessel-based molecular study using antibodies against vascular endothelial cells (CD31), the connective tissue growth factor or the vascular endothelial growth factor. We also found that these 3D imaging markers individually characterize the development of renal TI lesions or glomerulopathy, quantitative and integrated use of all of them provide more information for differentiating the two renal conditions. Our findings thus establish a practical strategy to characterize the CKD-associated renal injuries by the microangiography-based 3D imaging and highlight the impact of dysfunctional microvasculature as a whole on the pathogenesis of the renal lesions.

## Introduction

Chronic kidney disease (CKD) is a worldwide health issue. In spite of the high prevalence and incidence^1–3^, the pathogenic mechanisms underlying the development and progression of CKD, particularly their relation with the alteration of renal microvasculature, remain unclear. To overcome this obstacle hindering earlier diagnosis and more efficient treatment of CKD^4–8^, it is imperative to develop a better understanding of the structure of the renal microvasculature highly correlated with the pathogenesis underlying CKD^9, 10^. However, certain concerns regarding the procedure for diagnosing CKD including: (1) the pathophysiology-based abnormal microvasculature remains to be refined; (2) conventional pathological diagnosis depends mainly on 2D structures, but 3D imaging is recommended to profile the abnormal areas from the pathology perspective, including the fine lesions in the kidney^11, 12^; and (3) there is a risk of bleeding, infection or other complications in patients after undergoing renal biopsy^13–15^; need to be addressed.

Recently, we established a three dimensional (3D) microtomography platform based on synchrotron X-rays with greatly improved performance which is sufficient to resolve these above mentioned obstacles^16, 17^. Our imaging approach demonstrated previously the 3D profiling of entire microvascular networks and the neighboring fine structures in organs or tissues with micrometer resolution^18, 19^. The resolution achieved by our microtomography is sufficient to resolve 1 µm test patterns with a 20x, NA = 0.4 lens. (see Fig. S1, for example showing an X-ray image of a nanofabricated Au test pattern) with very fast imaging acquisition and processing. Our X-ray imaging platform also features nanoscale resolution and has been successfully applied to investigate tumor angiogenesis at tissue and cell level^20–26^. For this work specifically, the microtomography is suitable for exploring the kidney microvasculature^27–29^ and nanotomography supplemented the detailed examination.

Two complementary animal models of CKD were chosen for this study. The unilateral ureteral obstruction (UUO) model, representative of renal tubulointerstitial (TI) lesions showing dilated tubular lumens, atrophic tubular epithelial cells, leukocytic infiltration into the interstitium and extracellular matrix accumulation^30–33^. The focal segmental glomerulosclerosis (FSGS) model, concerning a specific type of glomerulopathy of which the main renal injury is typically located in the glomerulus affected^34–36^.

In addition, angiogenic cytokines, such as connective tissue growth factor (CTGF) and vascular endothelial growth factor (VEGF), are also involved in tissue repair, and other essential biological functions, including vascular changes and fibrosis in CKD^37, 38^. Both proteins are highly expressed in the renal lesions of CKD patients and have been shown to play a pathogenic role in this group of renal disorders. Furthermore, these two proteins are also required for the establishment of the microvasculature and the regulation of angiogenesis^39, 40^.

The purpose of this study was to seek unique renal microvessel-based microscopic 3D imaging features, capable of reflecting the status of pathological lesions observed in CKD patients. Five distinct image features were successfully identified by high resolution synchrotron microtomography strongly associated with renal TI lesions and/or glomerulopathy. We further characterize these 3D image features and validated them unique imaging markers suitable for differentiating the microvascular alterations between these renal lesions in a disease course manner.

## Results

### Complementary renal lesions in mouse UUO and FSGS models

**Figure 1:**
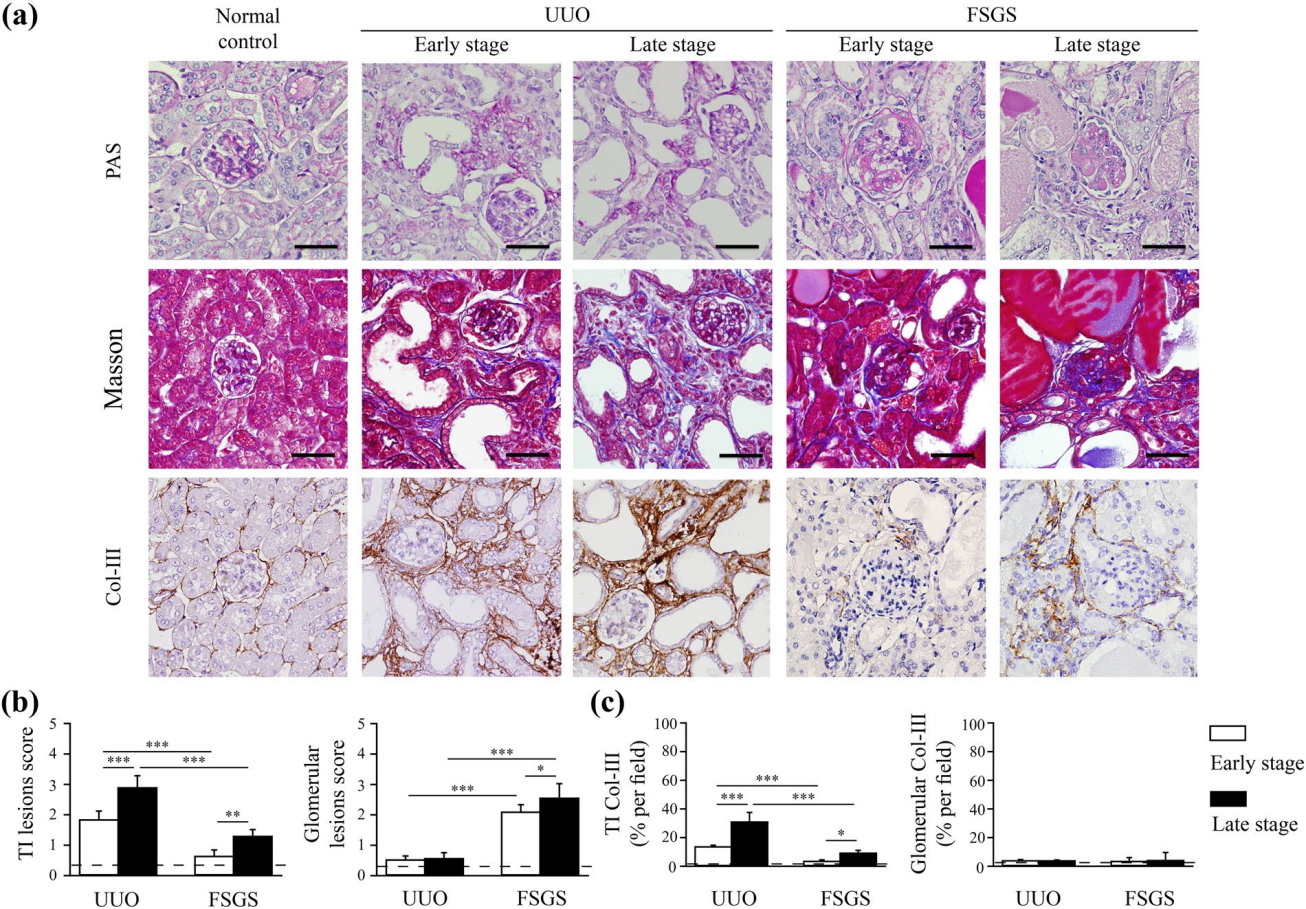
Complementary renal lesions in mouse UUO and FSGS models. (**a**) Renal histopathological evaluation by PAS, Masson’s trichrome and Col-III staining in the normal control, UUO early stage, UUO late stage, FSGS early stage and FSGS late stage mice. The original magnification was 400x each. Scale bar = 50 μm. (**b**) The quantitative results were shown as the TI lesions score and glomerular lesions score, respectively. (**c**) The quantitative results of Col-III positive area (% per field) in the renal TI lesions and glomerulus. The horizontal dashed line indicates the level of the normal control mice. UUO-unilateral ureteral obstruction; FSGS-focal segmental glomerulosclerosis; TI-tubulointerstitial; Col-III-collagen-III. n = 7 mice per group. *p < 0.05, **p < 0.01, ***p < 0.005.

Two mouse models of CKD, UUO that predominantly featured renal TI lesions and FSGS more in favor of glomerulopathy, were used to correlate the alterations in the microvasculature by simultaneous analysis with a high-resolution synchrotron-based X-ray imaging and tomography. As shown in Fig. 1a, in the early stage of UUO at day 7, light microscopy showed that the mice exhibited dilated Bowman’s spaces, ischemic collapse of the glomerular tufts, interstitial inflammation, and dilatation of renal tubular lumen with or without atrophic tubular epithelial cells in focal areas. For the late stage of renal lesions, marked fibrosis was evident and associated with persistently increasing tubular dilation and mononuclear leukocyte infiltration (Fig. 1a). However, in the FSGS mice, there were scattered but significant sclerotic areas in some glomeruli at day 14 and a large number of sclerotic areas in the glomeruli at day 28, but not as much renal TI lesions were observed compared with the UUO mice (Fig. 1a). The severity of renal TI lesions was further evaluated by “TI lesions score” for both UUO and FSGS mice. As shown in Fig. 1b, TI lesions score was significantly increased in the UUO mice at early stage and was persistently increased until the late stage of UUO mice compared with normal control mice (Fig. 1b). In contrast, there was no significant difference in TI lesions score at early stage of the FSGS mice compared with normal control mice, although TI lesions score was increased in the late stage of the FSGS mice compared with that of normal control and early stage FSGS mice (Fig. 1b). Meanwhile, the severity of glomerular lesions was further evaluated by “glomerular lesions score” for each group. As shown in Fig. 1b, compared with normal control mice, glomerular lesions score was increased early after the induction of FSGS, and the score was significantly increased over time until the late stage when the mice were sacrificed. In addition, the glomerular lesions score of each of early and late stages of FSGS mice was greatly increased compared with that of UUO mice (Fig. 1b).

In parallel, the severity of renal fibrosis was determined using immunohistochemistry (IHC) with collagen (Col)-III in both mouse models. As shown in Fig. 1c, in the renal TI lesions, the percentage of Col-III expression was greatly increased in the early stage of UUO mice compared with the normal control mice, and this effect persisted until the late stage of the UUO mice. In contrast, both in the early stage and late stage of FSGS mice, in the renal TI lesions, there was less in the percentage of Col-III expression compared with the UUO mice (Fig. 1c). However, the percentage of Col-III expression of the renal TI lesions was significantly increased in the late stage, but not in the early stage of FSGS mice compared with those of normal control mice (Fig. 1c).

### Reconstructed 3D renal microvasculature using synchrotron microtomography

**Figure 2:**
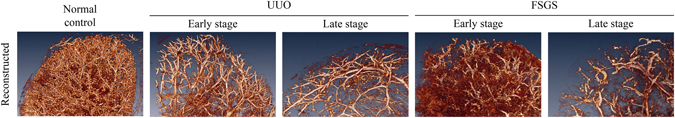
Reconstructed 3D renal microvasculature using synchrotron microtomography. Overview of the renal tomographic images from the normal control, UUO early stage, UUO late stage, FSGS early stage and FSGS late stage mice. UUO-unilateral ureteral obstruction; FSGS-focal segmental glomerulosclerosis. n = 7 mice per group.

**Figure 3:**
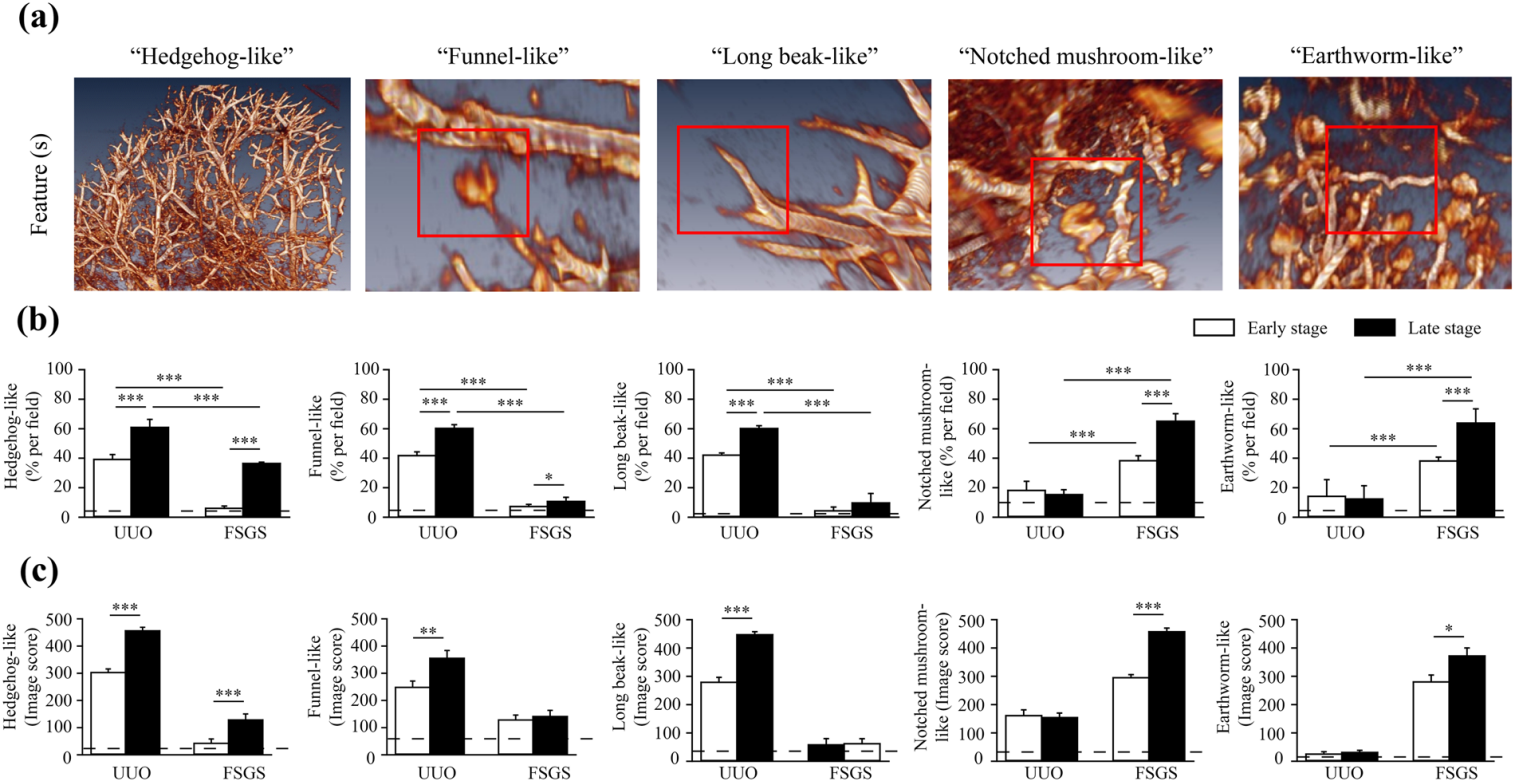
Characteristic 3D imaging markers distinguish early or late stages of the renal lesions in UUO and FSGS mice. (**a**) Five images were identified: hedgehog-like, funnel-like, long beak-like, notched mushroom-like and earthworm-like (each highlighted in square). (**b**) The quantitative results were shown as the percentage of hedgehog-like, funnel-like, long beak-like, notched mushroom-like and earthworm-like images per field. (**c**) Image score of hedgehog-like, funnel-like, long beak-like, notched mushroom-like and earthworm-like images for UUO or FSGS. The horizontal dashed line indicates the level of the normal control mice. UUO-unilateral ureteral obstruction; FSGS-focal segmental glomerulosclerosis. n = 7 mice per group. *p < 0.05, **p < 0.01, ***p < 0.005.

The pictures in Fig. 1a are used as an example; these images are typically 2D pictures that fail to reveal a stereo structure, such as that in a 3D picture, which often confers more comprehensive, insightful information in terms of highlighting fine structures, including microvascular structures. Moreover, based on our pilot studies, it is inevitable that a setback in the alignment of the individual histological pictures of the renal histopathology that make up the stereogram will be encountered. In the present study, high resolution microtomography was performed to assess the changes in the microvasculature in the UUO and FSGS mice. As shown in Fig. 2, after image reconstruction, there was a readily identifiable vascular system in the normal control mice, including the microvasculature in the cortical region of the kidney and relatively intact glomeruli that were evenly distributed in the cortex. However, a disrupted configuration and apparent rarefaction of the microvasculature in the cortex of the kidney and an extensive loss of glomeruli, which were relatively unevenly distributed within the renal cortex, were observed even at an early stage, day 7, in the UUO mice. In particular, there was an abrupt, discontinuous segment between the hilus of the glomerulus and the afferent arteriole, making the affected glomerulus appears as a funnel (Figs 2 and 3). At the late stage when the mice were sacrificed, almost all of the glomeruli disappeared, and this also contributed to the expanded spaces between the residual microvascular structures (Fig. 2). In contrast, we found that the entire renal vascular system including the microvasculature was clearly visible, with the exception of intense, focal, twisted microvascular profiles in the renal cortex, and there was random loss of glomeruli, in the early stage, day 14, in the FSGS mice (Fig. 2). However, in the late stage, day 28, in the FSGS mice, both rarefaction of the microvasculature in the cortex of the kidney and diffuse loss of glomeruli were observed (Fig. 2). Furthermore, the renal microtomography features of the FSGS mice did not show identifiable funnel-like glomeruli, which were observed in UUO mice at both the early and late stages of the model (Fig. 2).

### Characteristic 3D imaging markers distinguish early or late stages of the renal lesions in UUO and FSGS mice

Visual assessment of the imaging features has been essential for diagnostic applications. Detection of the gain or loss of these imaging features was routinely used as the criteria for the differentiation of disorders^41–43^. In our study, a comparative analysis was performed to establish potential 3D imaging features that can be associated with individual histopathological characteristics in the two complementary mouse models of renal inflammation and fibrosis. Our extensive search of the 3D high resolution synchrotron microtomography data of normal, UUO and FSGS mouse kidneys leads to five characteristic, distinctive 3D imaging markers were identified. We label these five markers tentatively as “hedgehog-like”, “funnel-like”, “long beak-like”, “notched mushroom-like” and “earthworm-like” (Fig. 3a) for their shapes. Specifically, (1) the hedgehog-like 3D image marker presented a diffuse loss of nearly all of the glomeruli, that could be attributed to the diffuse loss of contrast medium inside the interlobular arteries, the glomerular capillaries and renal interstitial capillaries, suggesting narrowing or obstruction of the capillary lumens (Fig. 3a); (2) the funnel-like marker indicated an abruptly discontinuous segment at the entry into the glomerulus and the afferent arteriole adjacent to the glomerulus, making the contracted glomerulus funnel-like (Fig. 3a); (3) the long beak-like marker showed the narrowing of the afferent arteriolar due to diffuse loss of glomerlulus and making the afferent arteriole “sharpened”, mimicking a long beak (Fig. 3a). Thus, these three particular imaging features reflect the renal histopathology (e.g., collapse of glomerular tufts, tubulointerstitial inflammation, extracellular matrix accumulation, urine retention in the tubular lumens and interstitial fibrosis), in which the interlobular arteries, arterioles of the hilus of the glomerulus, capillaries inside the glomerulus, and/or renal interstitial capillaries might encounter partial or complete obstruction during the development and/or progression of the disease course in the mouse models (Figs 1a and 3a).

The notched mushroom-like 3D imaging marker indicated a fovea-like defect in the glomerulus (likely attributed to the random ischemia of the glomeruli affected) (Fig. 3a). The earthworm-like marker represented deformed afferent arterioles, suggesting the tortuosity of the microvessels, including the afferent arterioles and interlobular arteries. These two imaging features could be associated with histopathological feature of glomerular sclerosis and renal interstitial capillaries fragility due to collagen depositions obstructing the blood flow during the disease development and progression (Figs 1a and 3a).

As shown in Fig. 3b, the UUO mice showed significantly higher percentages of the hedgehog-like, funnel-like and long beak-like 3D imaging marker than the FSGS mice at early stages. Similar images remained in the late stage when the mice were sacrificed. However, the notched mushroom-like and earthworm-like marker were observed early after the induction of FSGS, and their percentages increased over time until the late stage when the mice were sacrificed (Fig. 3b). In addition, the percentage of these 3D imaging markers in the FSGS mice increased significantly compared with those of UUO mice in the early stage, and this effect persisted until the late stage. These findings suggest that these five distinctive 3D imaging markers can indeed differentiate the altered microvascular structures between the renal TI lesions and glomerulopathy as represented by the mouse UUO and FSGS models.

We further performed a detailed analysis to correlate each of these 3D imaging markers with specific histopathological features of the kidney on the interlobular arteries, arterioles, capillary network of the glomerular tufts (composed of part of arterioles and abundant capillaries) and renal interstitium in both UUO and FSGS models (Figs 1a and 3a). To quantify of these imaging markers, an “image score” was derived using the formula for each specimen to describe the overall microvasculature condition. As shown in Fig. 3c, the image scores of hedgehog-like, funnel-like and long beak-like imaging markers in UUO mice were significantly higher on late stage than those of on early stage of renal disease. On the other hand, the image scores of the notched mushroom-like and earthworm-like imaging markers in FSGS mice were significantly higher on late stage than on early stage of renal disease.

### Distinguishing of the stages of renal lesions using an automatic 3D renal vessel tracing algorithm

**Figure 4:**
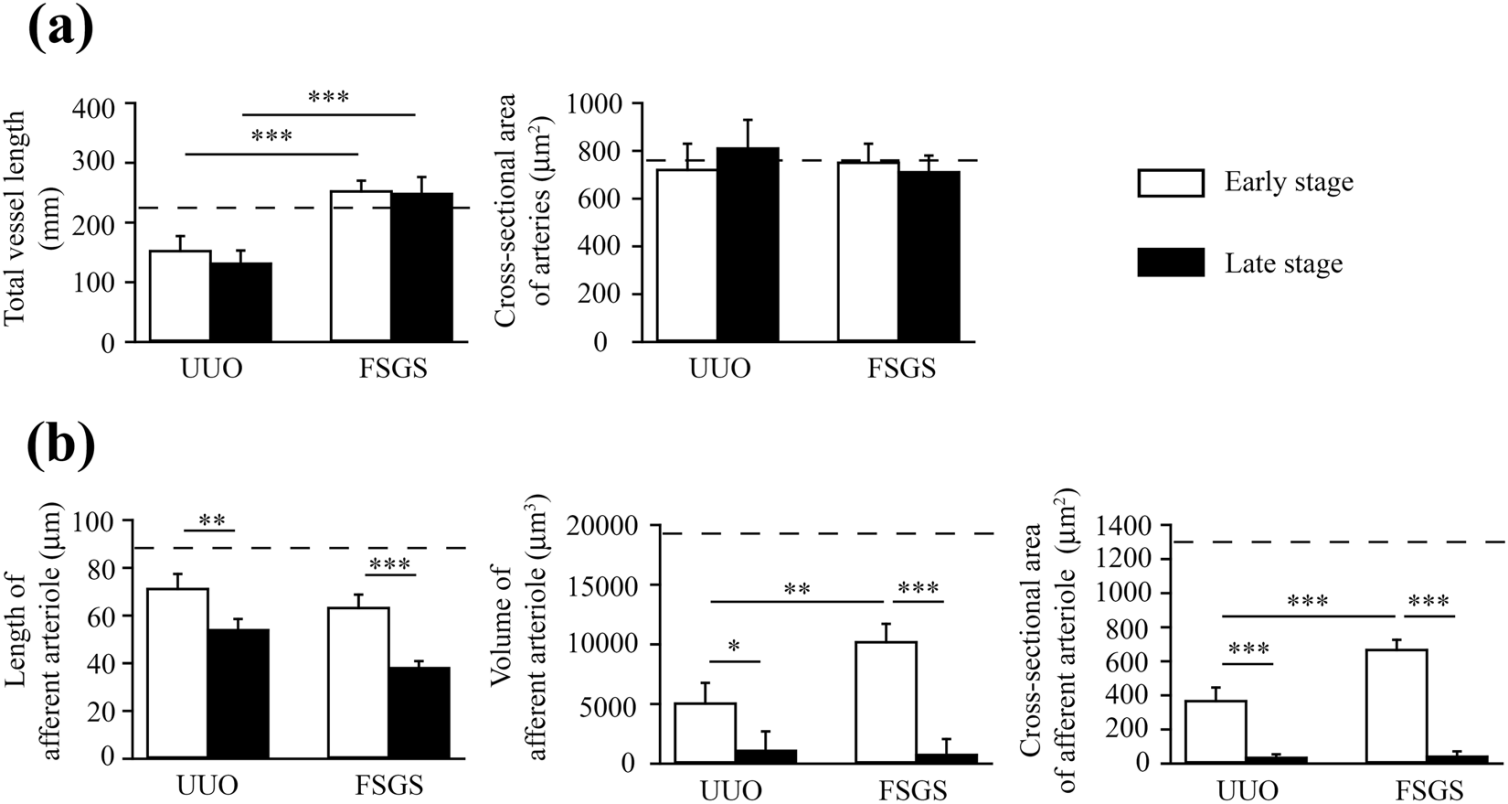
Distinguishing of the stages of renal lesions using an automatic 3D renal vessel tracing algorithm. (**a**) Total vessel length and cross-sectional area of arteries. (**b**) Length of afferent arteriole, volume of afferent arteriole and cross-sectional area of afferent arteriole. The horizontal dashed line indicates the level of the normal control mice. UUO-unilateral ureteral obstruction; FSGS-focal segmental glomerulosclerosis; TI-tubulointerstitial. n = 7 mice per group. *p < 0.05, **p < 0.01, ***p < 0.005.

In addition to determine the population of the 3D imaging markers, we also developed an automatic 3D tracing algorithm to quantify the other structure parameters in the kidney microvasculature. As shown in Fig. 4a, the total vessel length in the kidney was markedly reduced in UUO mice but not in the FSGS mice, the latter showing similar levels to normal control mice. No significant difference in the level of cross-sectional area of arteries identified in the whole kidney among these UUO, FSGS and normal control mice. Hence, we sought to specifically examine the arterioles of the glomerulus, and, as shown in Fig. 4b, the results showed significantly decreased levels of the length of afferent arteriole, volume of afferent arteriole and the level of cross-sectional area of the microvessel identified in the whole kidney in both the UUO and FSGS mice each compared with those of normal control mice. Furthermore, the levels of these parameters tended to decrease with time as demonstrated by their significantly reduced values in the late stage compared to those of the early stage in both models. In the early stage of disease, compared with FSGS mice, UUO mice had greatly reduced volume of afferent arteriole and cross-sectional area of afferent arteriole (Fig. 4b). Collectively, these results are consistent with those of the renal 3D imaging markers analyzed (Fig. 3b).

### The renal microvasculature as highlighted by the expression of CD31 (a vascular endothelial cell marker)

**Figure 5:**
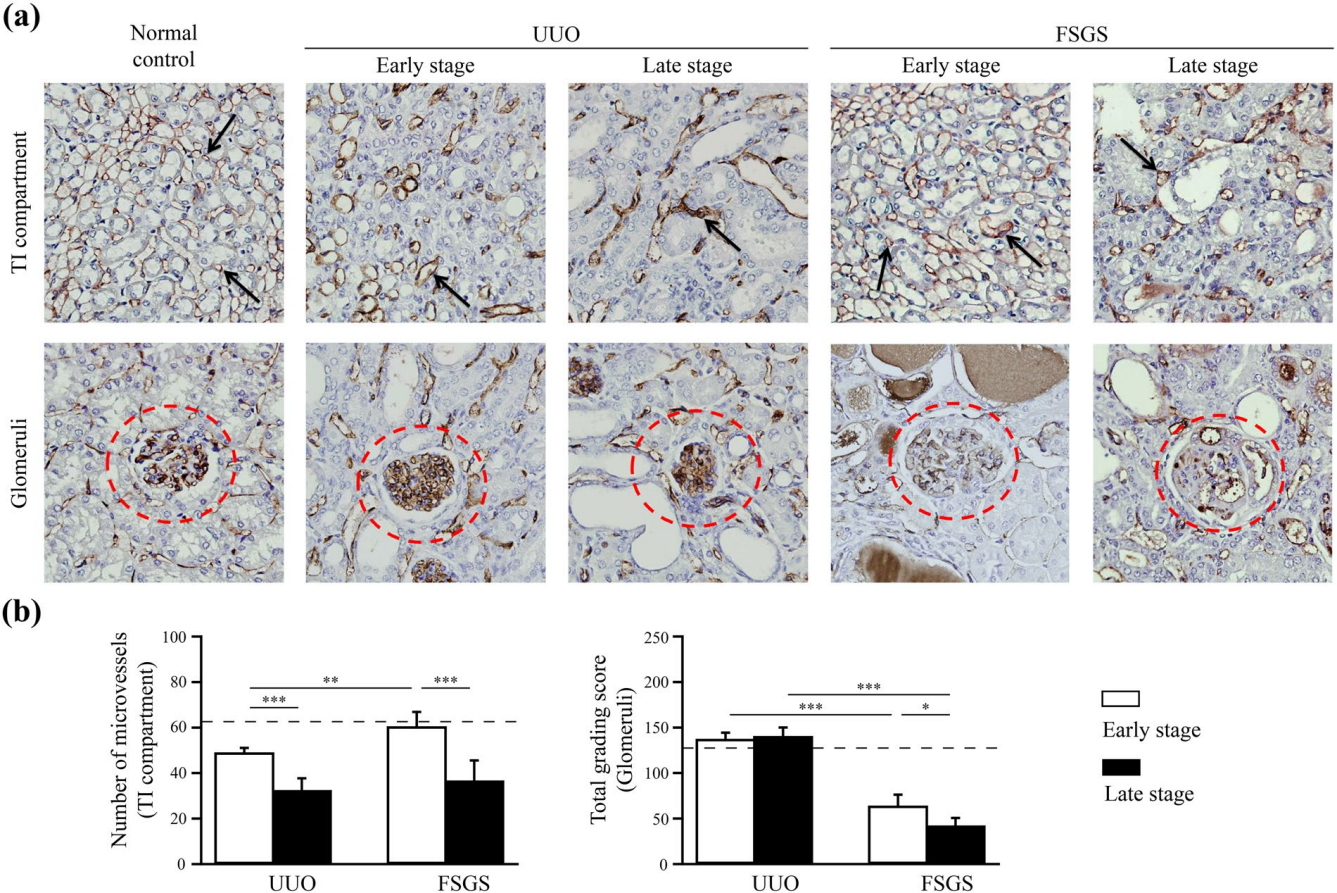
The renal microvasculature as highlighted by the expression of CD31 (a vascular endothelial cell marker). (**a**) The renal TI lesions and glomerulus (highlights in circle) microvasculature was stained with a CD31 antibody. Arrow indicates the positive staining for CD31 in the renal TI lesions. (**b**) The quantitative results were reported as the number of TI microvessels and total grading score. The horizontal dashed line indicates the level of the normal control mice. UUO-unilateral ureteral obstruction; FSGS-focal segmental glomerulosclerosis; TI-tubulointerstitial. n = 7 mice per group. *p < 0.05, **p < 0.01, ***p < 0.005.

To dissect the mechanistic pathways underlying the changes in the microvascular networks of the kidney and the capillaries of the glomerulus in UUO and FSGS mice, we assessed the distribution and quantity of vascular endothelial cells in renal tissue sections using IHC with CD31. As shown in Fig. 5, in the renal TI lesions, the number of microvessels was greatly decreased in the early stage UUO mice compared with the normal control mice, and this effect persisted until the late stage in the UUO mice. However, in the early stage of FSGS mice, the number of microvessels in the renal TI lesions was not reduced compared with the normal control mice, although it was decreased in the late stage of FSGS mice (Fig. 5).

The grading of CD31-positive glomeruli by IHC was calculated for each group. As shown in Fig. 5b, although there was no difference in total grading score between the UUO and normal control mice, the FSGS mice showed a significant decrease in total grading score of the disease compared with the normal control mice.

### Correlation between the expression levels of angiogenesis-related cytokines and the renal microvasculature

**Figure 6:**
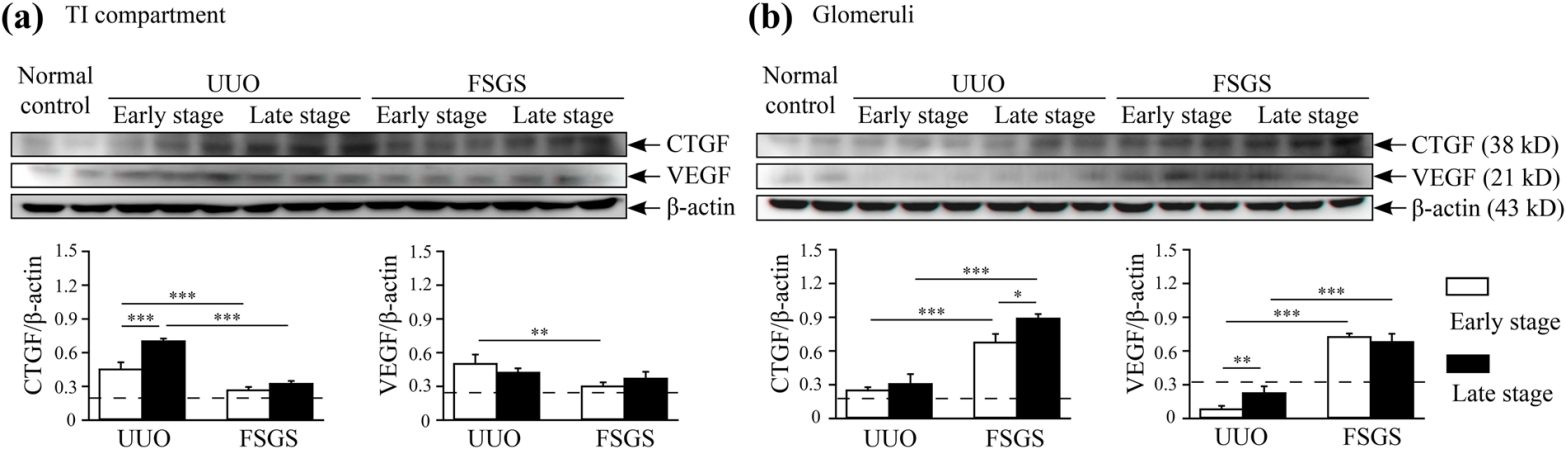
Correlation between the expression levels of angiogenesis-related cytokines and the renal microvasculature. (**a**) The levels of CTGF and VEGF proteins in the renal TI lesions. (**b**) The levels of CTGF and VEGF proteins in the glomeruli. β-actin was used as the internal control. The horizontal dashed line indicates the level of the normal control mice. UUO-unilateral ureteral obstruction; FSGS-focal segmental glomerulosclerosis; CTGF-connective tissue growth factor; VEGF-vascular endothelial growth factor; TI-tubulointerstitial. n = 7 mice per group. *p < 0.05, **p < 0.01, ***p < 0.005.

In addition to those 3D imaging markers, tissues from the glomeruli or the renal TI lesions were collected by laser capture microdissection (LCM) to further investigate the pathogenic mechanisms. In the renal TI lesions, the renal CTGF levels were significantly increased in both the early and late stages of the disease in UUO mice compared with normal control mice (Fig. 6a). The renal VEGF levels were also significantly increased in both the early and late stage in UUO mice compared with normal control mice. In contrast, there was no significant difference in the levels of CTGF and VEGF in the renal TI lesions between the FSGS and normal control mice at the early stage. Although there was significantly increased at the late stage. In the glomerulus, the renal CTGF levels were increased in both the early and late stages of disease in UUO mice compared with normal control mice (Fig. 6b). However, the renal VEGF levels were significantly decreased in the early stage in UUO mice compared with normal control mice. In contrast, FSGS mice showed significantly increased levels of CTGF and VEGF in the glomeruli compared with normal control mice, and persistently higher renal levels of these two proteins were observed in the late stage in FSGS mice (The original full-length blots were shown in Supplementary Fig. S2).

## Discussion

Synchrotron sources have been providing very bright X-ray beams for decades critical to obtain high-resolution 3D images with high throughput^44–46^. In this study, we take advantage specifically the very high resolution and demonstrated that the performance is essential for our objective to characterize the microvasculature alternation due to CKD. We successfully identified a number of 3D imaging markers characteristic to the altered microvessels in the mouse kidneys. We also explored the potential implications of these markers in renal TI lesion and glomerulopathy using two mouse UUO and FSGS models of human CKD by comparing them with renal histopathology features and the results of renal microvessel-based molecular analysis. These 3D imaging markers were therefore firmly established capable of characterizing differential renal lesions in these distinct experimental models of CKD. Note these differential renal lesions are often observed in CKD patients and these 3D imaging markers are potentially applicable to human biopsy specimens.

The key performance factor of tomography required to distinguish and characterize these image markers is the excellent 3D resolution provided by the synchrotron microtomography. The diameter of renal microvessels, such as glomerular capillaries, arterioles, and interlobular arteries, varies at a range of 40 μm to less than 2 μm, thus many small renal microvessels were not detected in previous lab-based micro CTs studies^47–51^. The high resolution 3D kidney microvasculature delineated by synchrotron X-ray microtomography, on the other hand, allows us to perform adequate quantitative analysis to determine valuable structural and morphological parameters. These high precision and high fidelity 3D structural parameters further enable our study of the minute modification of renal microvascularture^52–55^. As a benefit of the superior performance, for example, that the number of microvessels determined by our imaging method was significantly higher than that of lab-based micro CTs^56^.

**Figure 7:**
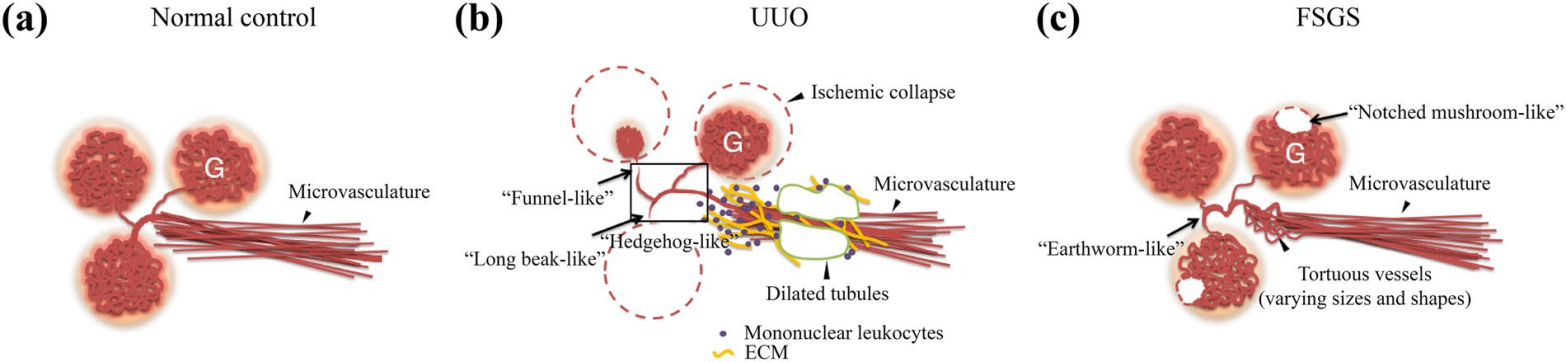
Hypothesized pathogenic pathways reflected by the images in the two complementary models of chronic kidney disease. (**a**) The normal control mice display relatively intact glomeruli that were evenly distributed in the cortex. (**b**) The UUO (mainly renal TI lesions) mice showed a disrupted configuration and apparent rarefaction of the microvasculature, which was displayed in the hedgehog-like (highlights in square), funnel-like and long beak-like images. (**c**) In FSGS (mainly glomerulopathy) mice, the microvasculature was clearly visible, with the exception of intense, focal twisted microvascular profiles in the renal cortex and the random defects of glomeruli, which are represented by the notched mushroom-like and earthworm-like imaging markers. These schematic diagrams were constructed according to the imaging features and reflected the pathological findings (PAS and IHC staining). UUO-unilateral ureteral obstruction; FSGS-focal segmental glomerulosclerosis; TI-tubulointerstitial; G-glomerulus; ECM-extracellular matrix.

Synchrotron microtomography results of normal control mouse kidney microvasculature, Figs 2 and 7a, show straight and uniform microvessels around the glomeruli. In UUO mouse kidneys, the hedgehog-like 3D image markers, in contrast, are associated with reduction in the number of microvessels, the funnel-like markers denote a discontinuous connection between the efferent arteries and glomerulus, and the long beak-like markers delineate constricted microvessels abutting the hilus of the glomerulus (Fig. 3a). Comparing these 3D imaging markers in UUO mice with the histopathology of each of the renal components, they can be attributed to mononuclear leukocyte infiltration, extracellular matrix accumulation and/or urine retention in the tubular lumens. These factors alone or in combination can yield pressure increase from outside the microvessels and contribute to either partial or complete obstruction of blood flow along the microvasculature, thus reducing the volume of blood in the glomerulus (Figs 1a,3a and 7b).

Regarding the markers found in the FSGS mice, the notched mushroom-like markers indicate defects in the glomerulus and the earthworm-like markers denote deformed microvessels of varying sizes and shapes, characteristic to the FSGS model. Furthermore, these 3D imaging markers in the FSGS mice can be attributed to the sclerosis of the microvessels inside the glomerulus or those in the renal interstitial compartment (Figs 1a,3a and 7c). These results suggest that our imaging findings indeed can be associated with the histopathological profiles of the microvascular network-related alterations/changes in the evolution of CKD.

To further clarify these structural alterations which were not observed before, we also investigated the molecular pathways underlying the anomaly of the renal microvessels in both mouse models and linked the findings to the 3D imaging markers with CTGF and VEGF, both of which are angiogenesis-associated cytokines that are necessary for the development and homeostasis of the renal microvasculature^39, 40^. Our data clearly showed that compared with normal control mice, FSGS mice showed increased levels of CTGF and VEGF in the glomeruli. Whereas, this effect was inhibited in UUO mice, which had relatively lower levels of both proteins compared with FSGS mice. The results suggest that FSGS mice may exhibit specific damage to the capillary endothelial cells of the glomeruli (as adriamycin, the inducer for this mouse model, is relatively toxic to vessels of various sizes), and this effect may be reflected by the notched mushroom-like marker observed in the FSGS mice (Figs 1a,3a and 6b).

In this regard, although the presence of hedgehog-like markers suggesting the diffuse effacement of the glomeruli in UUO mice, this alteration could be secondary to the partially or completely obstructed microvessels in the renal TI lesions or afferent arterioles, rather than direct damage to the endothelial cells in the glomerular tufts of the mice. Consistent with this notion, the UUO mice mainly showed a long beak-like marker in the renal TI lesions, as suggested by the increased CTGF and VEGF protein levels in a clinical-course-dependent manner on both day 7 and 14 specimens. Additionally, this effect in the renal TI lesions could contribute to the formation of the funnel-like marker in the kidneys of the UUO mice (Figs 1a,3a and 6a).

Note that synchrotron microtomography studies of kidneys in mouse models were reported elsewhere^57, 58^, these studies were not able to establish imaging markers to register microvessel alterations. Furthermore, the previous works lack a comparative analysis of the 3D images and the expression levels of endothelial cell-based molecular biomarkers to further delineate the pathophysiological pathway involved in the pathogenesis of renal inflammation and the subsequent progression of the renal condition to fibrosis, the key histopathological hallmarks of CKD.

Our study not only demonstrated with practical examples and robust analysis the importance of high resolution synchrotron microtomography, but also establish a set of candidate imaging markers that reflects the status and changes in the microvascular network during the different courses and progressions of renal inflammation/fibrosis that simulate those of CKD patients. Noted that light microscopy can generate 3D images in serial tissue sections on a morphological basis^59–61^, however, it is very difficult to align the reconstructed adjacent images to properly reflect the morphological features. The required serial sectioning of specimens for 3D imaging cannot reach the resolution level demonstrated with high resolution synchrotron microtomography without heavy investment of time in sectioning and imaging thin specimens. No 3D image markers were reported by such approach, either.

On the other hand, the five 3D imaging markers we identified in this study working in combination can predict the status of renal TI lesions and glomerulopathy, based on the alterations in the microvasculature in the kidney. In addition, we propose that an integrated used of all these 3D markers of the microvessels and renal pathology, including both histology and the distribution of the microvessels by an immunohistochemical study of the endothelial cells using CD31 antibodies, would prove to be a strategy that could be used in further investigations of the pathogenesis of renal injury in CKD and help better elucidate the fundamental mechanistic pathways underlying the development and progression of CKD in which the microvessels in the kidney serve as a key compartment that were regulated before the development of the various types of CKD.

## Materials and Methods

### Ethical statement

Mice were purchased from the National Laboratory Animal Center and housed in the National Defense Medical Center (NDMC), Taiwan. The mice were kept in individual ventilated cages and kept at 25 ± 2 °C on a 12-hour light/dark cycle. The experimental procedures were approved by the Animal Protection Association (APA) of the Republic of China (ROC) and the NDMC Institutional Animal Care and Utilization Committee (IACUC). In addition, the number of animals used was approved by an ethical committee at the Ministry of Science and Technology of the Republic of China (MOST 103-2320-B-016-011-MY3). The experiments were performed in such a way that the number of animals used was minimized. We have confirmed that all methods were performed in accordance with the relevant guidelines and regulations.

### Induction of the UUO and FSGS models in mice

UUO was induced in 8-week-old BALB/c male mice^62, 63^. In brief, the mice were subjected to left ureter ligation at the junction with 6–0 silk suture under anesthesia with an intramuscular injection of Zoletil 50 (50 mg/kg; Virbac Laboratories, Carros, FR). First, the back skin of the mice was shaved and the abdominal wall was cut on the back of the mice. A short suture line was dragged underneath the ureter and then tied with three knots. After the operation, a subcutaneous injection of Flunixin (2.5 mg/kg; Norbrook Laboratories, Northern Ireland, UK) was administered as an analgesic agent. The mice were sacrificed at day 7 or 14 after induction.

FSGS model was induced in 8-week-old male BALB/c mice by intravenous injection of a single dose of adriamycin (12.5 mg/kg; Pfizer, New York, USA)^64, 65^. The mice were sacrificed at day 14 or 28 after induction.

### Pathological evaluation

PAS staining was performed for the renal pathological evaluation. Renal tissues were fixed in 10% buffered formalin immediately after removal. After approximately 24 hours, the samples were placed in an automatic tissue processor (Sakura Finetek, Tokyo, JP) and dehydrated. Then, the samples were embedded in paraffin using a paraffin-dispenser (Sakura Finetek). Two-micrometer-thick renal sections were cut with a sliding microtome (Leica, Wetzlar, DE) and were attached onto glass slides. The slides were then placed in a 75 °C incubator for 30 minutes and immersed in xylene for 10 minutes. The sections were rehydrated using a gradient alcohol series of 100%, 95%, 85%, and 70%, and the sections were finally placed in deionized water. The slides were then immersed in 0.5% periodic acid solution, placed in Schiff reagent and stained with Mayer’s hematoxylin. Masson’s trichrome staining was performed on formalin-fixed paraffin-embedded renal sections. First, the sections were stained in Weigert’s iron hematoxylin and then immersed in Biebrich scarlet-acid fuchsin solution. Next, the sections were immersed in phosphomolybdic-phosphotungstic acid solution and then transferred to aniline blue solution. Finally, the sections were immersed in acetic acid solution and dehydrated in 95% ethyl alcohol.

Renal TI lesions were evaluated by the severity of the tubular dilation, interstitial fibrosis, and mononuclear cell infiltration, as previously described^66, 67^. In brief, the TI lesions score was semi-quantitatively determined from 0 to 4+ in an average of 5 fields per kidney section (0, no changes; 1, changes affecting less than 25% of the section; 2, changes affecting 25 to 50% of the section; 3, changes affecting 50 to 75% of the section; and 4, changes affecting 75 to 100% of the section). For the glomeruli, glomerular lesions were evaluated by the percentage of glomeruli presenting sclerotic lesions, as previously described^68, 69^. In brief, the glomerular lesions score was semi-quantitatively determined from 0 to 4+ in an average of 10 glomerulus per kidney section (0, no changes; 1, changes affecting less than 25% of the glomeruli; 2, changes affecting 25 to 50% of the glomeruli; 3, changes affecting 50 to 75% of the glomeruli; and 4, changes affecting 75 to 100% of the glomeruli).

### IHC

For IHC, formalin-fixed paraffin-embedded renal sections (2 μm) were stained with biotin-labeled goat anti-Col-III (Southern Biotech, Alabama, USA) or rat anti-CD31 (BD) antibodies overnight at 4 °C, washed with TBST, and incubated with a horseradish peroxidase (HRP)-conjugated rabbit anti-goat IgG or rabbit anti-rat IgG (Dako, Glostrup, DK) antibody for 1 hour. The sections were then visualized with 3, 3′-diaminobenzidine (Dako) and counterstained with hematoxylin.

The percentage of Col-III expression in the renal TI lesions or glomeruli was automatically quantified using the Pax-it software (Midwest Information Systems, Iowa Avenue, USA). To highlight the alterations in the microvessels, the distribution of CD31 (an endothelial cell marker) in the microvessels was automatically quantified using the Pax-it software (Midwest Information Systems). After the area of the microvessels in the renal TI lesions was identified, individual microvessels were counted and presented as number of TI microvessels within a 400 × field (12.533 mm^2^ per field), as previously described^69, 70^. Microvessels in the sclerotic or fibrotic areas and areas closely adjacent to the tubular lumens were not included in the vessel counts. The grading of the CD31-positive glomeruli was determined as previously described, with slight modification^71, 72^. In brief, 40 glomeruli were examined on each slide, and the positive staining was assigned a score ranging from 0 to 3+ (0, positive staining less than 25% in the cross section of a single glomerulus; 1, positive staining between 25 to 50% in the cross section of a single glomerulus; 2, positive staining between 50 to 75% in the cross section of a single glomerulus; 3, positive staining more than 75% in the cross section of a single glomerulus); the total grading score was calculated for each specimen using the following equation: total grading score = (% glomeruli grading negative × 0) + (% glomeruli grading 1 + × 1) + (% glomeruli grading 2 + × 2) + (% glomeruli grading 3 + × 3). The values ranged from 0 to a maximum of 300.

### Preparation of renal samples

UUO or FSGS mice were sacrificed with an intramuscular injection of Zoletil 50 (Virbac) as previously described^73^. The kidneys were washed under physiological perfusion pressure with heparinized saline (400 IU/ml) and then injected with a contrast agent as previously described^19^. In this study, barium sulfate particles were administrated to the kidney vessels via a syringe, attached to the manual pump, and injected at a rate of 1 μl/s^18, 21, 58^. Barium sulfate particles were filtered by 0.5 μm filter cup to remove aggregated barium sulfate. Then, barium sulfate particles were washed with distilled water and centrifuged at 3000 *g* for 60 minutes. Finally, barium sulfate particles were suspension in phosphate buffer saline (PBS) at the concentration of 30% (w/w). The filtered barium sulfate particles were suspended in the solution without aggregation and thus able to reach the smallest vessels. After fixation, the samples were washed with PBS solution for 1 hour, dehydrated with a gradient of ethanol solutions 3 times, and embedded in Embed-812 (Electron Microscope science, Hatfield, UK) as previously described^73^. Finally, the samples were incubated in a 65 °C oven for 8 hours for polymerization.

### 3D analysis of tomography reconstructed imaging

The experiments were performed on the TLS 01 A beamline of the National Synchrotron Radiation Research Center (NSRRC, Hsinchu, Taiwan). Unmonochromatized X-rays (4 keV to 30 keV, with peak intensity at ~12 keV) emitted by a 4.5 tesla superconducting wavelength shifter of the 1.5 GeV electron storage ring operated at a top-up mode at a constant current of 360 mA^74^. Two 550 µm silicon wafers were used to attenuate the strong X-rays to an estimated dosage of 33.9 Gy. The exposure time was 100 ms per image. The distance between sample and scintillator was 5 cm. We used a 2 × lens for overviews of the whole kidney and 5x lens to perform the local tomography; the pixel size in the final image was ~2.86 µm (2x lens) and ~1.14 µm (5x, NA = 0.21). The resolution degraded accordingly from the best case shown in Fig. 1S obtained with a 20x lens (NA = 0.4), a reasonable trade-off for a larger field of view.

The tomographic reconstruction using filtered back projection algorithm was performed on sets of 1,000 images at equal angular spacing within 180 degrees with a code written with the IDL software. For the tracing analysis, the 3D data were first segmented based on their voxel values and then the skeleton were traced with the “centerline tree” and “auto skeleton” functions in the “skeletonization package” of the Amira 6.0 software^74^. All the 3D visualization tasks were processed also with the Amira 6.0. These 3D synchrotron microtomographic imaging markers were then quantitatively analyzed and presented as percentages by calculating the numbers of each imaging marker in a total of 100 randomly selected glomeruli with adjacent microvascular structures, and the imaging profiles between the two mouse models were compared.

### Quantification of the synchrotron microtomographic imaging markers

Each of the five 3D synchrotron microtomographic imaging markers was rated as grade 0 to 5 scores, which depends on the degree of defects on interlobular arteries, arterioles of the hilus of the glomerulus, capillaries inside the glomerulus, and/or renal interstitial capillaries affected. The image score was then determined according to the following formula for each specimen: image score = (% “image marker grade 0” × 0) + (% “image marker grade 1” × 1) + (% “image marker grade 2” × 2) + (% “image marker grade 3” × 3) + (% “image marker grade 4” × 4) + (% “image marker grade 5” × 5). The values ranged from 0 to a maximum of 500.

### 3D renal vessel tracing algorithm

The tracing algorithm was based on the procedures: first, the image voxels were segmented into several connected clusters, which were defined if every voxel in the cluster was connected at least neighbor one. Second, image voxels in a connected cluster were encoded based on the idea of region-growth method. Every voxels in this connected cluster was encoded by a number representing the sequential distance of the shortest path from the origin. A codelet i was defined by voxels with coding number i−1, i, i + 1 and its center of mass was calculated based on all of these voxels. The tracing for one connected cluster was performed by simply following the codelets sequentially with increasing indices through all the encoded voxels. Finally, all of the encoded voxels were traced. Based on the rules, total vessel length, cross-sectional area of arteries, length of afferent arteriole, volume of afferent arteriole, cross-sectional area of afferent arteriole and other relevant quantitative parameters for the 3D imaging markers were then calculated.

### LCM and Western blot analysis

The renal tissues were embedded in OCT and immediately stored at −20 °C. The samples were placed in the pre-cooled cryostat for sectioning, and the 8-μm-thick sections were spread onto Arturus PEN membrane glass slides (ABI, Massachusetts, USA). The staining protocol was then performed according to the instructions that were provided with the HistoGene® LCM Frozen section staining kit (ABI). In brief, the slides were fixed in 75% ethanol for 30 seconds and washed with distilled water for 30 seconds. The HistoGene® staining solution (ABI) was then dropped onto the sections, incubated for 20 seconds, and washed with distilled water for 30 seconds. The sections were dehydrated using a gradient of alcohol solutions, 75%, 95%, and 100% ethanol, and the slides were then immersed in xylene. The desired cells or areas were selected using specialized microdissection software, ArcturusXT™ LCM System (ABI). The proteins were then purified from the samples and used for western blot analysis. Each protein sample was separated on a 10% SDS-PAGE gel and blotted onto 0.22 μm Immobilon-PSQ transfer membranes (Millipore, Darmstadt, DE). The membranes were then incubated in blocking buffer (Tris-buffered saline containing 5% skim milk) for 1 hour at room temperature and incubated with rabbit antibodies against CTGF, VEGF or β-actin (Santa Cruz Biotechnology, Texas, USA) overnight at 4 °C. Next, the membranes were incubated with an HRP-conjugated goat anti-rabbit IgG antibody (Santa Cruz) for 1 hour at room temperature; the proteins were then detected with Chemiluminescent Reagent Plus (PerkinElmer Life Sciences, Massachusetts, USA), and images were captured on the UVP system (Biospectrum, California, USA).

### Statistical analysis

The definition of these five 3D imaging markers extracted from the microtomography image data was performed by two radiologists and two renal pathologists. Statistical analysis was performed with IBM SPSS Software 20 (IBM, Armonk, New York, USA). The results are presented as the mean ± SEM. Comparisons among groups were performed using one-way ANOVA, with post hoc correction by Tukey’s method. A *p* value less than 0.05 was considered statistically significant.

## Electronic supplementary material

Supplementary Information
