# Supplementary material for: Differential synchrotron X-ray imaging markers based on the renal microvasculature for tubulointerstitial lesions and glomerulopathy
Source: Sci Rep. 2017 Jun 14;7:3488. doi: 10.1038/s41598-017-03677-x (PMC5471266; doi:10.1038/s41598-017-03677-x)
Supplement: Supplementary file 1 — Supplementary Information [file 41598_2017_3677_MOESM1_ESM.pdf]

Supplementary Information

Differential synchrotron X-ray imaging markers based on the renal microvasculature for tubulointerstitial lesions and glomerulopathy

Yu-Chuan Lin[1], Yeukuang Hwu[2], Guo-Shu Huang[3], Michael Hsiao[4], Tsung-Tse Lee[2], Shun-Min Yang[2], Ting-Kuo Lee[2], Nan-Yow Chen[5], Sung-Sen Yang[6], Ann Chen[1, 7*] and Shuk-Man Ka[1, 8*]

[1]Graduate Institute of Life Sciences, National Defense Medical Center, Taipei, Taiwan

[2]Institute of Physics, Academia Sinica, Taipei, Taiwan

[3]Department of Radiology, Tri-Service General Hospital, National Defense Medical Center, Taipei, Taiwan

[4]Genomics Research Center, Academia Sinica, Taipei, Taiwan

[5]National Center for High-Performance Computing, Hsinchu, Taiwan

[6]Division of Nephrology, Department of Internal Medicine, Tri-Service General Hospital, National Defense Medical Center, Taipei, Taiwan

[7]Department of Pathology, Tri-Service General Hospital, National Defense Medical Center, Taipei, Taiwan

[8]Graduate Institute of Aerospace and Undersea Medicine, Academy of Medicine, National Defense Medical Center, Taipei, Taiwan

***Correspondence to:**

Ann Chen, MD, PhD; E-mail: annchen31717@gmail.com

Or

Shuk-Man Ka, PhD; E-mail: shukmanka@gmail.com

Supplementary Figure S1

Supplementary Figure S2

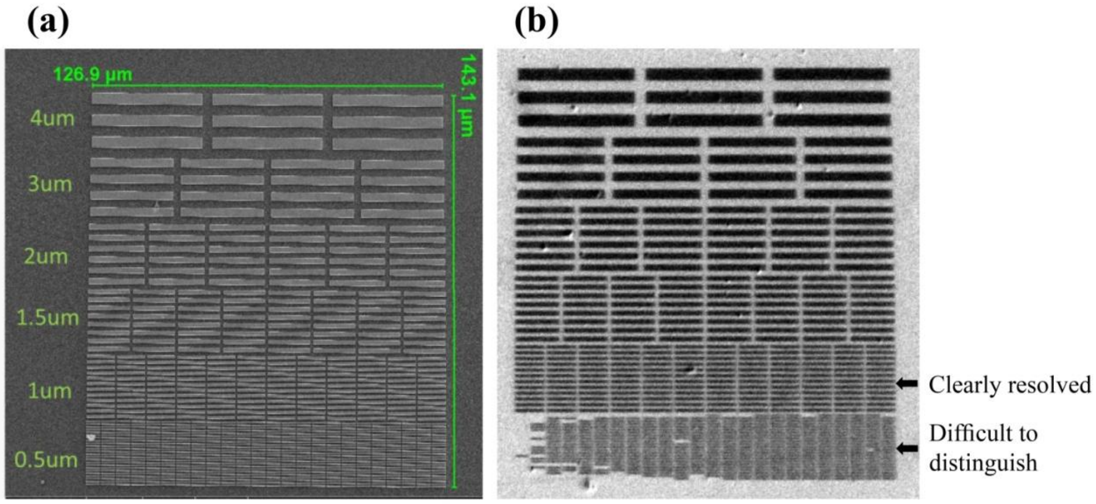

Supplementary Fig. S1. The resolution of 3D synchrotron microtomography. (a) Scanning electron micrograph of a nanofabricated Au test pattern shows the smallest feature of 0.5 μm. **(b)** X-ray micrograph of the test pattern shows 1 μm feature can be clearly resolved with a 20x, numerical aperture (NA) = 0.4 lens.

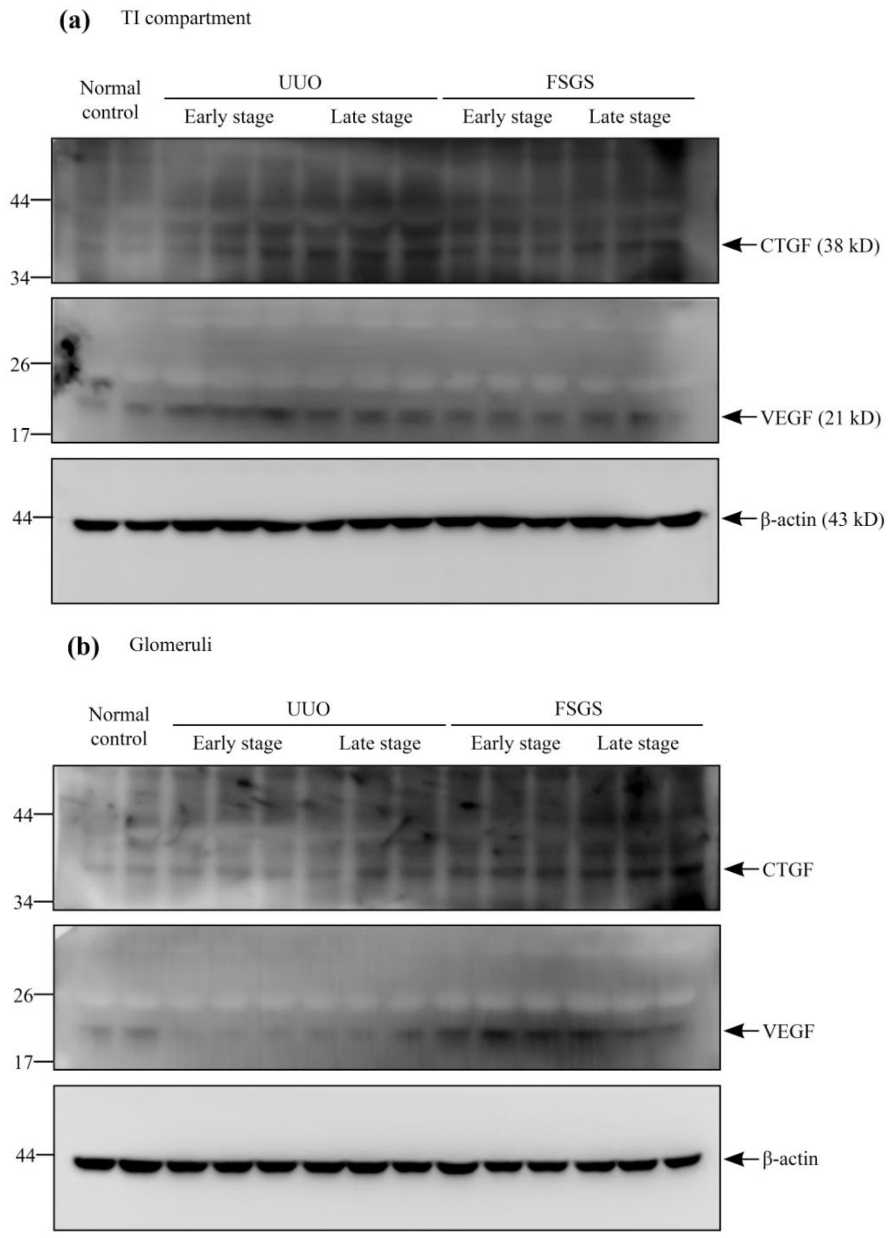

Supplementary Fig. S2. The original full-length blots for the expression levels of angiogenesis-related cytokines in the renal microvasculature. (a) The levels of CTGF and VEGF proteins in the renal TI lesions. **(b)** The levels of CTGF and VEGF proteins in the glomeruli. UUO-unilateral ureteral obstruction; FSGS-focal segmental glomerulosclerosis; CTGF-connective tissue growth factor; VEGF-vascular endothelial growth factor; TI-tubulointerstitial.
